# Comparative Analysis of Two NGS-Based Platforms for Product-of-Conception Karyotyping

**DOI:** 10.3390/genes15081100

**Published:** 2024-08-21

**Authors:** Yuri Murase, Yui Shichiri, Hidehito Inagaki, Tatsuya Nakano, Yoshiharu Nakaoka, Yoshiharu Morimoto, Tomoko Ichikawa, Haruki Nishizawa, Eiji Sugihara, Hiroki Kurahashi

**Affiliations:** 1 Division of Molecular Genetics, Center for Medical Science, Fujita Health University, 1-98 Dengakugakubo, Kutsukake-cho, Toyoake, Aichi 470-1192, Japan; 2 IVF Namba Clinic, Osaka 557-0013, Japan; 3 HORAC Grandfront Osaka Clinic, Osaka 530-0011, Japan; 4 Department of Obstetrics and Gynecology, Nippon Medical School, Tokyo 113-0083, Japan; 5 Department of Obstetrics and Gynecology, Fujita Health University School of Medicine, Aichi 470-1192, Japan; 6 Center for Joint Research Facilities Support, Research Promotion and Support Headquarters, Fujita Health University, Aichi 470-1192, Japan; 7 Department of Clinical Genetics, Fujita Health University Hospital, Aichi 470-1192, Japan

**Keywords:** product of conception, NGS, karyotype, X chromosome, methylation

## Abstract

Cytogenetic information about the product of conception (POC) is important to determine the presence of recurrent chromosomal abnormalities that are an indication for preimplantation genetic testing for aneuploidy or structural rearrangements. Although microscopic examination by G-staining has long been used for such an evaluation, detection failures are relatively common with this method, due to cell-culture-related issues. The utility of low-coverage whole-genome sequencing (lcWGS) using short-read next-generation sequencing (NGS) has been highlighted recently as an alternative cytogenomic approach for POC analysis. We, here, performed comparative analysis of two NGS-based protocols for this purpose based on different short-read sequencers (the Illumina VeriSeq system using a MiSeq sequencer and the Thermo Fisher ReproSeq system using an Ion S5 sequencer). The cytogenomic diagnosis obtained with each NGS method was equivalent in each of 20 POC samples analyzed. Notably, X chromosome sequence reads were reduced in some female samples with both systems. The possibility of low-level mosaicism for monosomy X as an explanation for this was excluded by FISH analysis. Additional data from samples with various degrees of X chromosome aneuploidy suggested that it was a technical artifact related to X chromosome inactivation. Indeed, subsequent nanopore sequencing indicated that the DNA in the samples showing the artifact was predominantly unmethylated. Our current findings indicate that although X chromosome data must be interpreted with caution, both the systems we tested for NGS-based lcWGS are useful alternatives for the karyotyping of POC samples.

## 1. Introduction

Miscarriage is the most common complication of early pregnancy, with an occurrence rate of 10–15% among clinically recognized pregnancies [1,2]. Most clinical miscarriages occur in the first trimester, and more than half are due to chromosomal abnormalities [3]. Some of the affected couples undergo recurrent pregnancy loss, and this accounts for 1–3% of all pregnancies [3]. Since recurrent pregnancy loss has heterogenous etiologies, cytogenetic testing of the product of conception (POC) is important for these couples to determine the cause of the miscarriages. This occasionally leads to proposals to conduct preimplantation genetic testing (PGT) for embryo selection prior to any subsequent pregnancies. The most common chromosomal abnormality to cause a miscarriage is autosomal trisomy due to age-related meiotic error, the frequency of which increases with advanced maternal age [3]. Polyploidy and monosomy X are the second- and third-leading causes of miscarriage. Other chromosomal anomalies that have been implicated in cases of recurrent risk of pregnancy loss are unbalanced structural abnormalities related to parental balanced translocations or inversions. Since these types of miscarriages could potentially be avoided by embryo selection via PGT for aneuploidy or structural rearrangements (PGT-A or PGT-SRs), it is important to identify cytogenetic abnormalities in the POCs of at-risk couples.

Screening for chromosomal abnormalities in POC samples has long been conducted using conventional G-banding. This approach has practical limitations, however, that are related to its requirement for cell culture, including possible fetal demise, the preferential growth of maternal decidua cells, and the artifactual emergence or disappearance of abnormal chromosomes during cell growth [4,5]. In contrast, DNA-based methodologies, such as SNP microarray and array comparative genomic hybridization (aCGH), do not require cell culture and have become alternative techniques to G-banding for the cytogenetic analysis of POC samples, although their cost is considerably higher [6,7]. Furthermore, next-generation sequencing (NGS) has also become a powerful and low-cost DNA-based cytogenomic technique via the quantitative analysis of the number of sequence reads. Low-coverage whole-genome sequencing (lcWGS) has been shown to be useful in aneuploidy screening for PGT-A and PGT-SR and for cytogenomic POC analysis [8,9,10].

The Illumina VeriSeq system using the MiSeq sequencer and the Thermo Fisher ReproSeq system that uses the Ion S5 sequencer are two major NGS platforms for cytogenomic analysis in PGT-A/PGT-SR. The performance of these two systems has been reported to be comparable for whole-genome amplification (WGA) samples from embryo biopsies, with a data concordance of more than 99% [11]. To the best of our knowledge, however, there has been no comparative study to date of the performance of these two systems in POC karyotyping. We, here, conducted this comparison to understand the utility of these systems in POC karyotyping. In addition, we frequently observed decreased sequence reads of the X chromosome in both systems, which was finally revealed to be a technical artifact specific to an NGS-based cytogenomic study that arises from the heterochromatinization of an inactivated X chromosome in female samples. We discussed the origin of this artifact and called attention to its impact on the interpretation of X chromosome copy numbers.

## 2. Materials and Methods

### 2.1. Samples

Chorionic tissue sampling was performed after first-trimester miscarriages that arose following assisted reproductive technology (ART) interventions. Chorionic tissues were washed with phosphate buffer solution to remove blood clots. After the maternal decidua was carefully removed under microscopy, the chorionic villi were separated by trained laboratory staff. A total of 20 samples were obtained for further chromosome analysis. The mean age of the pregnant women in the study population was 37 years (range 28–43 years), and the mean gestational age at miscarriage was 8.9 weeks (range 6.4–10.4 weeks). The research protocol used in this study was approved by the local ethics committee of Fujita Health University, Japan (HG21-033). We also retrospectively analyzed four POC samples with various X chromosome aneuploidies, as well as a positive X chromosome artifact. Written informed consent was obtained from all the participating couples in this study who miscarried.

### 2.2. lcWGS by NGS

The karyotypes of the POC samples were determined by lcWGS in accordance with two different protocols using short-read NGS. Genomic DNA was extracted from chorionic villi using a Gentra Puregene Tissue Kit (Qiagen Inc., Valencia, CA, USA) in accordance with the manufacturer’s protocol. Whole-genome amplification (WGA) was performed using the SurePlex WGA Kit (Illumina, San Diego, CA, USA) as per the manufacturer’s instructions. Nextera libraries were prepared from the WGA-amplified DNA and subsequently sequenced with a VeriSeq PGS assay system by MiSeq (Illumina). The sequencing data were analyzed using BlueFuse Multi analysis software v4.5. The karyotype reports were produced manually.

The same POC samples were also analyzed using another short-read NGS method with Ion Chef equipment and the Ion S5 sequencer (Thermo Fisher Scientific, Waltham, MA, USA). The genomic DNA extracts from the POC samples were subjected to WGA using an Ion Reproseq PGS kit (Thermo Fisher Scientific). Template preparation and chip loading were conducted using the Ion Chef system, as described in the manufacturer’s instructions. The chip was then loaded and sequenced on an Ion S5 XL sequencer. The acceptance values used for quality control (QC) parameters for both the entire run and individual samples were as previously reported [12]. Karyotype reports were produced via the IonReporter system with Japanese translation by OVUS Inc. (Nagoya, Japan).

For samples with pronounced X chromosome artifacts, a second round of genomic DNA extraction was performed from the chorionic villi using the same Gentra Puregene Tissue Kit (Qiagen) with minor modifications to the protocol. Briefly, the samples were treated with proteinase K at 55 °C overnight (originally for 1 h), incubated for protein aggregation for 1 h (originally for 10 min), and centrifuged for protein precipitation at 4 °C (originally carried out at room temperature).

### 2.3. QF-PCR

QF-PCR analysis was performed on three of our study cases with alleged 46,XX karyotypes to examine for the possible presence of triploidy. This analysis was performed as previously described [10]. Briefly, the Aneufast QF-PCR kit was used in accordance with the manufacturer’s protocol (Genomed Diagnostics AG, Altendorf, Switzerland). All PCR products were genotyped using a SeqStudio Genetic Analyzer (Thermo Fisher Scientific) with GeneMappe software 6 (Thermo Fisher Scientific).

### 2.4. FISH

We carried out interphase FISH analysis using chorionic villi cells to analyze for mosaic monosomy X using FISH probes specific for the X or the Y chromosome included in the AneuVysion Multicolor DNA Probe Kit (Abbott, Abbott Park, IL, USA). To detect low-level mosaicism, 100 interphase nuclei were analyzed.

### 2.5. Methylation Study

Nanopore sequencing was used for the quantification of methylated cytosine at specific loci on the X chromosome. Briefly, 3.0 µg of DNA was used to generate a library with the V14 ligation sequencing kit (SQK-LSK114) in accordance with the manufacturer’s protocol (ONT, London, UK). Sequencing was performed by a GridION sequencer, a high-accuracy model, at a 400 bps setting. After loading the library to GridION R10.4.1 flow cells, we obtained about 16 Gb of targeted sequences. For adaptive sampling, we prepared the following FASTA files of the X chromosome with the T2T CHM13 reference (whole chromosome). Since it is recommended that 3–4% of the whole genome be designated using a FASTA file for adaptive sampling, we used the short arm of the X chromosome for the quantification of methylation. Modified base calling and alignment were carried out using dorado 0.4.3, with a model DNA_r10.4.1_e8.2_400bps_hac@v4.2.0. After sorting the aligned data with SAMtools 1.18, we obtained a bam file with 5-methylcytosine modification information. The status of X chromosome inactivation (XCI) was evaluated using the average methylation frequency by calculating the ratio of the methylated/unmethylated alleles [13]. The upstream region of the *SLC9A7* gene, located in a region previously shown to well reflect XCI, as evidenced by methylation data, was examined [14].

## 3. Results

**Figure 1:**
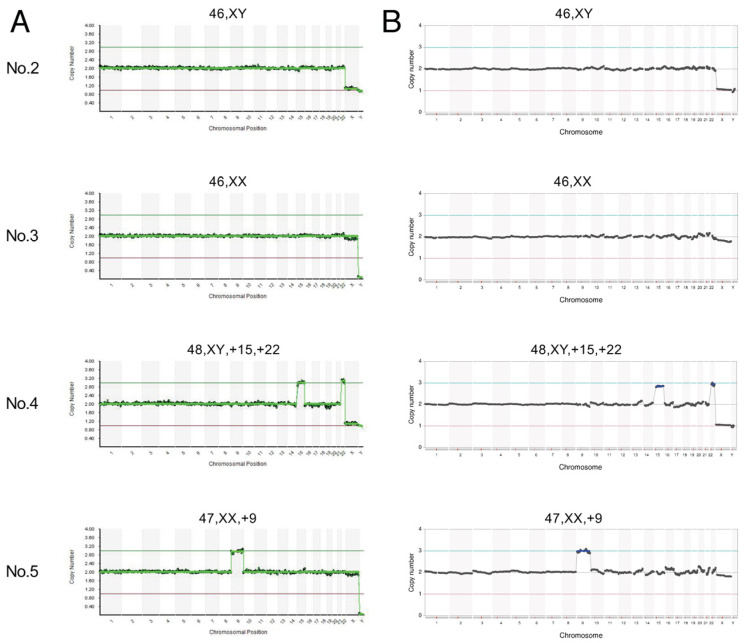
Representative aneuploidies detected among the POC samples. Comparison of the data chart obtained using the Illumina VeriSeq system with a MiSeq sequencer (**A**) and using the Thermo Fisher ReproSeq system with an Ion S5 sequencer (**B**). Sample numbers are indicated on the left, while predicted karyotypes are indicated on the top.

NGS-based lcWGS was performed for 20 POC samples. Two cytogenomic analysis methods, the Illumina VeriSeq system using a MiSeq sequencer and the Thermo Fisher ReproSeq system using an Ion S5 sequencer, were applied to all POC samples (Figure 1). The results are summarized in Table 1. We detected chromosomal abnormalities in 12 cases, with the remaining 8 samples showing a normal karyotype. Of the 12 abnormal cases, 11 showed autosomal aneuploidy, while 1 showed monosomy X. Nine cases showed aneuploidy for a single autosome, while two samples showed multiple aneuploidies involving two or more chromosomes (cases 4 and 13). No autosomal monosomy was observed, while monosomy X was detected in cases 13 and 18, the former of which also carried multiple autosomal trisomies. In our eight allegedly normal diploid samples, we observed five females, so the ratio was slightly biased toward females. To exclude 69,XXX triploidy cases, we performed QF-PCR to distinguish between 69,XXX and 46,XX. QF-PCR results indicated all five female cases were true 46,XX. The final cytogenetic diagnosis for each sample was identical for both systems.

**Figure 2:**
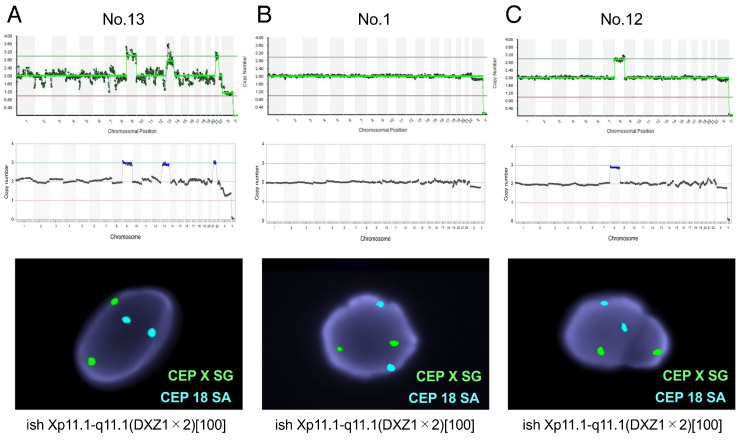
X chromosome FISH. Comparison of the data chart obtained using the Illumina VeriSeq system with a MiSeq sequencer (**upper** panel) and using the Thermo Fisher ReproSeq system with an Ion S5 sequencer (**middle** panel). Lower panels present FISH results with centromere probes for the X chromosome (green) and chromosome 18 (blue). Two X chromosome centromeric signals were detected in all 100 cells analyzed in each of the study cases despite the decrease in the number of X chromosome read counts. Sample numbers are indicated on the top. (**A**) Case 13. (**B**) Case 1. (**C**) Case 12.

Both analysis systems identified monosomy X mosaicism in one sample (case 13; Figure 2A, upper and middle panels). To confirm this diagnosis, we performed interphase FISH analysis using an X centromere probe (Figure 2A, bottom). In observations of 100 cells for case 13, all the interphase nuclei showed two signals for the X chromosome centromere. When we re-examined the cytogenomic charts of all samples manually by eye, most of the female samples showed a 10–20% reduction in sequence reads in the X chromosome (Figure 2B,C, upper and middle panels). The levels of these reductions were comparable between the two systems. When the number of X chromosomes was then examined by interphase FISH analysis, no monosomy X cells were detected in these samples (Figure 2B,C, bottom). These results suggested that the reduced X chromosome sequence reads in the female samples might have been due to technical artifacts.

**Figure 3:**
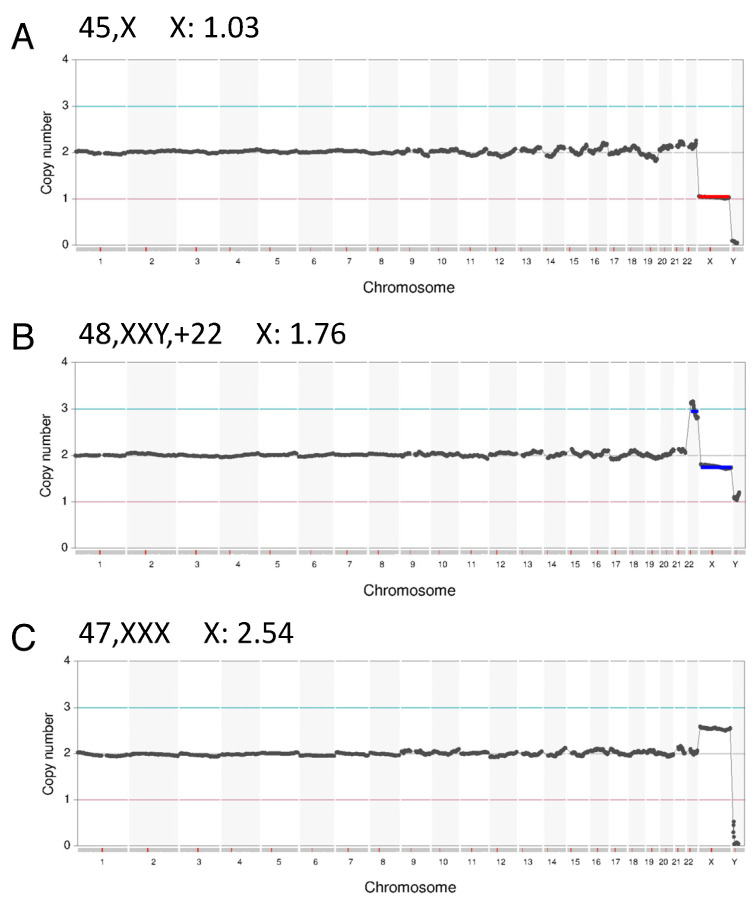
Reduction in X chromosome read counts in X chromosome aneuploidy. Data charts obtained with the Thermo Fisher ReproSeq system using an Ion S5 sequencer are shown. The predicted karyotype and X chromosome read counts are indicated on the top of each chart. (**A**) Case with 45,XX. (**B**) Case with 48,XXY,+22. (**C**) Case with 47,XXX.

Since a reduction in X chromosome sequence reads was observed only in female samples, we speculated that the mechanism underlying this artifact might be associated with X chromosome inactivation. To test this, we retrospectively analyzed POC samples with various X chromosome aneuploidies (Figure 3). The 45,X samples did not show this artifact at all, whereas XXY male samples showed the artifact in a similar manner to 46,XX female specimens. Notably, 47,XXX samples also showed high levels of the artifact. Since two X chromosomes are generally inactivated in a 47,XXX female, these results, indeed, suggested an association of the artifact with X chromosome inactivation.

**Figure 4:**
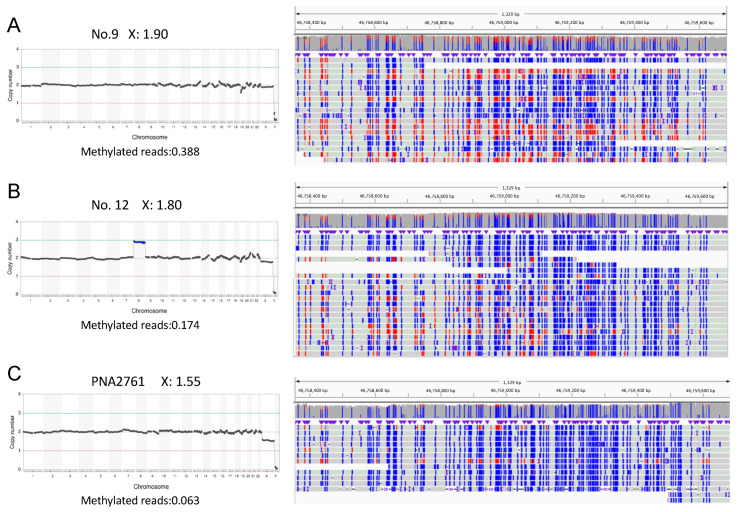
Methylation status of XCI-sensitive regions. The methylation status of XCI-sensitive regions was analyzed using a nanopore sequencer. Representative results for the upstream region of the *SLC9A7* gene (chrX:46758356-46759683) are shown. Red bars indicate 5mC of the CpG, whereas blue bars indicate mainly the non-methylated CpGs. (**A**) Case 9, with a less prominent artifact. (**B**) Case 12, with a higher degree of artifact. (**C**) Sample PNA2761, with the strongest artifact level obtained retrospectively. PNA2761 was finally found to be whole-chromosome uniparental isodisomy. The X chromosome read counts are indicated on the top of each chart, while the ratios of the methylated reads to the total reads are indicated at the bottom.

One of the X chromosomes in females is inactivated through the formation of a dense packaged heterochromatin structure, and it is not unreasonable to speculate that such inactivated heterochromatinized DNA would be resistant to DNA extraction or WGA, leading to a reduced number of sequence reads. XCI is generally mediated via epigenetic modification, with the increased methylation of the CpG islands at the promoter region of the genes sensitive to this process. We examined the methylation status of the extracted DNA from the POC samples using a nanopore sequencer. Decreased methylation levels were observed in samples that showed the artifact (case 12, Figure 4A,B). We then retrospectively analyzed POC samples showing the artifact more prominently and detected a robust reduction in methylated DNA (Figure 4C). The artifact level thus appeared to be correlated to reduced DNA methylation. These data prompted us to extract DNA using more stringent conditions, but this did not reduce the occurrence of the artifact at the X chromosome.

## 4. Discussion

We previously demonstrated the utility of NGS-based lcWGS for the cytogenetic testing of a miscarried fetus. We previously also analyzed a total of 300 POC samples using this method and identified chromosome abnormalities in 201 (67.0%) of those specimens [10]. Despite the small sample size of this comparative study of two different NGS methodologies, an abnormal karyotype was identified in 60% of the cases, while the remaining 40% of the cases showed a normal karyotype. The cytogenetic results from these two methods were identical. The detection rate for an abnormal karyotype was also similar to that previously reported using conventional G-banding methods [3], indicating that both approaches are similarly useful for POC karyotyping. In our previous study, we incorporated QF-PCR to detect polyploidy or genome-wide uniparental isodisomy. In this study, we found no instances of triploidy or genome-wide uniparental isodisomy, which we did detect in our previous study. It is noteworthy in this respect, however, that all our present POC samples were derived from ART pregnancies, and an intracytoplasmic sperm injection followed by microscopic confirmation of fertilization through the observation of male and female pronuclei was not likely to result in any triploidy or genome-wide uniparental isodisomy for such a limited number of samples. A combination of QF-PCR might be more useful than simple lcWGS alone in cases involving natural conception.

Cytogenomic analysis methods without the requirement for cell culture are important because culture failures can often arise due to fetal demise or the predominant growth of contaminated maternal decidua cells [4,5]. Furthermore, in instances of mosaic cytogenetic abnormalities, cells with abnormal chromosomes are often lost in culture via negative selection, thus affecting the detection of low-level mosaicism [15,16]. In the follow-up of PGT-A, and as mosaic embryos can generally be transferred without any appreciable risk, highly sensitive methods that can detect low-level mosaicism might be required when conducting prenatal diagnosis or in POC samples. In such situations, even if additional cell counts in a traditional karyotyping are recommended, the reliability of the detection of low-level mosaicism is limited [17]. Cytogenomic analysis without culture would be expected to improve this detection sensitivity.

Our data suggest that the reduced sequence reads from the X chromosome in female samples might be an artifact related to XCI, as this process involves the inactivation of one X chromosome through the formation of heterochromatin structures that may be resistant to proteolysis during genomic DNA extraction. This would necessarily then lead to a lower DNA yield from the inactivated X chromosome. Another possibility is that as the lcWGS protocol includes WGA, the efficiency might be affected by heterochromatin as a result of a low yield of the WGA product from the inactivated X chromosome. Our nanopore sequencing demonstrated that the decreased methylation levels in the female samples showing the artifact in the reads of X chromosome and the levels of the artifacts correlated to the levels of the reduction in methylated DNA. Since genomic DNA is used for nanopore sequencing without passing through WGA, the possibility that the inefficiency of WGA is causing the decreased read counts from the inactive X chromosome can be excluded. It is still possible that DNA extraction is inhibited by the heterochromatinization of an inactivated X chromosome. However, we could not determine this, since a re-extraction of DNA under more stringent conditions did not reduce the artifact level or the methylation bias. Elucidation of the mechanism underlying this artifact deserves further investigation.

In a clinical setting, attention needs to be called to the diagnosis of monosomy X mosaicism. While monosomy X is one of the most frequent cytogenetic abnormalities in the POC, the frequency of its mosaicism is still unknown. It is well established that 99% of embryos with monosomy X undergo spontaneous abortion. It is also well acknowledged that most of the Turner syndrome patients with a 45,X karyotype harbor a cryptic mosaicism of monosomy X, with the presence of a small number of normal cells, resulting in subclinical mosaicism [18,19]. These data suggest the possibility that monosomy X mosaicism is actually not a true cytogenetic abnormality of fetal demise. Indeed, it might occasionally be a contamination of maternal cells as a result of using methods that require cell culture or may sometimes be the artifact due to XCI. A more thorough analysis of low-level mosaicism in monosomy X would help to further validate these possibilities. Hence, the interpretation of instances of reduced X chromosome sequence reads is also a critical issue in genetic counseling related to an NGS-based prenatal diagnosis or preimplantation genetic testing for aneuploidy.

In conclusion, two cytogenomic analysis methods involving the Illumina VeriSeq system with a MisSeq sequencer and the Thermo Fisher ReproSeq system using an Ion S5 sequencer showed complete concordance in the karyotyping of POC samples. Importantly also, since the Ion Reporting system can automatically produce a karyotype report, it may have increased utility in the future for standard cytogenetic testing in a diagnostic laboratory, not only for POC samples, but also for chorionic villi samples or amniocentesis undergoing general screening tests, and for testing following mosaic embryo transfer in PGT-A or in NIPT-positive pregnancies.

## Figures and Tables

**Table 1:** Karyotypes of the analyzed POC samples using the two NGS-based methods.

	Results of Chromosomal Analysis by NGS	
Case No.	Illumina	Thermo Fisher Scientific
1	46,XX	46,XX
2	46,XY	46,XY
3	46,XX	46,XX
4	48,XY,+15,+22	48,XY,+15,+22
5	47,XX,+9	47,XX,+9
6	47,XY,+13	47,XY,+13
7	47,XY,+15	47,XY,+15
8	47,XY,+21	47,XY,+21
9	46,XX	46,XX
10	47,XX,+15	47,XX,+15
11	47,XY,+15	47,XY,+15
12	47,XX,+8	47,XX,+8
13	49,XX,+9,+13,+21/48,X,+9,+13,+21	49,XX,+9,+13,+21/48,X,+9,+13,+21
14	46,XY	46,XY
15	47,XY,+16	47,XY,+16
16	47,XY,+9	47,XY,+9
17	46,XX	46,XX
18	45,X	45,X
19	46,XX	46,XX
20	46,XY	46,XY

## Data Availability

The raw data supporting the conclusions of this article will be made available by the authors on request.
